# Association Between Predeployment Optimism and Onset of Postdeployment Pain in US Army Soldiers

**DOI:** 10.1001/jamanetworkopen.2018.8076

**Published:** 2019-02-08

**Authors:** Afton L. Hassett, Joseph A. Fisher, Loryana L. Vie, Whitney L. Kelley, Daniel J. Clauw, Martin E. P. Seligman

**Affiliations:** 1 Chronic Pain and Fatigue Research Center, University of Michigan, Ann Arbor; 2 Positive Psychology Center, University of Pennsylvania, Philadelphia; 3 Research Facilitation Laboratory/Army Analytics Group, Monterey, California

## Abstract

**Importance:**

Pain after deployment is a major health care concern. While risk factors have been previously studied, few studies have explored protective factors.

**Objective:**

To examine the prospective association between predeployment optimism and the onset of new pain after deployment in US Army soldiers.

**Design, Setting, and Participants:**

This prospective longitudinal cohort study examined US Army soldiers (active duty, Reserve, and National Guard) who deployed to Afghanistan or Iraq between February 12, 2010, and August 29, 2014, and completed the necessary psychological and health assessments before and after deployment. Analyses were performed in the Person-Event Data Environment between July 2016 and November 2018. This study relied exclusively on existing, secondary Army data. Of the 413 763 Army soldiers who met the specified deployment criteria, 385 925 soldiers were missing 1 or more of the required assessment forms. Of the remaining 27 838 soldiers who were examined for eligibility, 7104 soldiers were excluded because of preexisting back pain, joint pain, or frequent headaches. These exclusions resulted in a final analytic sample of 20 734 eligible soldiers.

**Main Outcomes and Measures:**

This study examined new reports of pain after deployment, including new back pain, joint pain, and frequent headaches.

**Results:**

Among 20 734 US Army soldiers (87.8% male; mean [SD] age, 29.06 [8.42] years), 37.3% reported pain in at least 1 new area of the body after deployment: 25.3% reported new back pain, 23.1% reported new joint pain, and 12.1% reported new frequent headaches. As a continuous measure, each 1-U increase in optimism was associated with 11% lower odds of reporting any new pain after deployment, even while adjusting for demographic, military, and combat factors (odds ratio, 0.89; 95% CI, 0.86-0.93). Tertile analyses revealed that compared with soldiers with high optimism (lowest odds of new pain) soldiers with low optimism had 35% greater odds of reporting new pain in any of the 3 sites evaluated (odds ratio, 1.35; 95% CI, 1.21-1.50). In addition, a larger increase in risk of new pain was observed when comparing the moderate-optimism and low-optimism groups rather than the high-optimism and moderate-optimism groups.

**Conclusions and Relevance:**

Higher levels of optimism were associated with lower odds of reporting new pain after deployment, over and above other common determinants of pain, including demographic and military characteristics and combat experiences. Soldiers with low levels of optimism before deployment could benefit from programs geared toward enhancing optimism.

## Key Points

**Question:**

Are higher levels of predeployment optimism among US Army soldiers associated with fewer reports of new pain after deployment?

**Findings:**

Among 20 734 US Army soldiers in this longitudinal cohort study, optimism was associated with 11% lower odds of reporting new postdeployment pain, even while adjusting for demographic, military, and combat factors, including traumatic experiences and combat injury. In addition, 37.3% of soldiers reported pain in at least 1 new area of the body after deployment.

**Meaning:**

The findings suggest that soldiers with low levels of predeployment optimism may be at greater risk of developing new postdeployment pain and may benefit from scalable interventions designed to increase optimism.

## Teaser

This longitudinal cohort study examines the association between predeployment factors and the onset of new pain after deployment in US Army soldiers.

## Introduction

Pain affects more Americans than coronary heart disease, diabetes, and cancer combined, at an estimated cost of $635 billion per year.^1^ Yet, the consequences of chronic musculoskeletal pain may be even more profoundly experienced in military personnel after deployment.^2^ For example, military musculoskeletal injuries result in more than 1 million medical encounters each year, and musculoskeletal and connective tissue disorders are the most common reason for medical evacuation of deployed personnel.^2,3^

Studies^4,5,6,7,8^ of veterans who served in Operation Enduring Freedom, Operation Iraqi Freedom, or Operation New Dawn have shown that from 40% to more than 80% report experiencing chronic pain after deployment. More than half of these soldiers describe pain that is moderate to severe^6,8^ occurring predominantly in the back, legs, shoulders, neck, and head.^5,7^ Such pain is frequently reported as lasting longer than 1 year, with more than half experiencing pain almost every day, if not constantly.^6^ Chronic pain in veterans is associated with other significant problems, such as functional disability, vocational limitations, family discord, greater health care use, traumatic brain injury, and psychiatric comorbidities, including posttraumatic stress disorder (PTSD), major depressive disorder, and substance abuse that includes opioid misuse.^5,8,9,10,11,12,13^

Determining who might be at risk for chronic pain after deployment is essential. Previous studies have shown that in soldiers deployed to Afghanistan and Iraq the following characteristics are associated with postdeployment pain: older age (>30 years),^6^ being married or previously married,^6^ exposure to combat^6,14^ (especially injury during combat^6^), duty involving heavy physical labor,^6^ and PTSD and other psychiatric conditions.^6,15^ Demographic and combat factors are rarely modifiable, and PTSD and other psychiatric comorbidities tend to be the sequelae of deployment^16^ rather than useful predeployment determinants of the development of pain. By reframing the question to instead assess what potentially modifiable predeployment characteristics are associated with fewer instances of postdeployment pain, new targets for treatment may be identified.

Although traumatic deployment events experienced during combat often precede PTSD,^17,18^ depression and substance abuse,^19,20^ and reports of postdeployment physical symptoms, including pain,^21^ these stressful experiences do not always result in negative outcomes. Many individuals are resilient to the potentially deleterious effects of combat, despite negative exposures. Therefore, it is important to understand what modifiable factors protect these soldiers from persistent negative outcomes following deployment, such as the development of chronic pain. One promising protective factor to explore is optimism. Optimism has been found to be a significant determinant of a wide range of positive physical health outcomes,^22,23,24^ including decreased pain sensitivity,^25^ enhanced conditioned pain modulation,^26^ lower risk for the development of chronic postsurgical pain,^27^ and better overall quality of life in adults and children with chronic pain.^28^ Among active duty soldiers, greater baseline levels of optimism have recently been linked to lower odds of being diagnosed as having PTSD, depression, anxiety, and adjustment disorders over a 2-year follow-up period.^29^

Much of the previous research evaluating postdeployment health concerns has been limited by the use of cross-sectional data from small studies of treatment-seeking veterans. Herein, the Person-Event Data Environment, a secure and comprehensive Army cloud-based data repository and analysis platform,^30,31^ was used to explore the overall prevalence of postdeployment pain and to investigate optimism as a determinant of the onset of new postdeployment pain in a robust sample of soldiers. Therefore, the objectives of this study were (1) to evaluate the incidence of new postdeployment pain in a large sample of soldiers who served in Operation Enduring Freedom, Operation Iraqi Freedom, or Operation New Dawn and (2) to assess whether having high levels of optimism before deployment is associated with a decreased likelihood of reporting new pain after deployment. We hypothesized that optimism would buffer the often deleterious effects of deployment and be related to decreased odds of reporting new pain, accounting for combat and other deployment experiences.

## Methods

This study followed the Strengthening the Reporting of Observational Studies in Epidemiology (STROBE) reporting guidelines. The study examined a subset of US Army active duty, Reserve, and National Guard soldiers who deployed to Afghanistan or Iraq between February 12, 2010, and August 29, 2014, for more than 1 day and no longer than 15 months. Additional study inclusion criteria were as follows: (1) they completed the Global Assessment Tool, a self-report questionnaire assessing psychosocial functioning taken annually by soldiers,^32,33^ in the year before their deployment (and indicated through an electronic “opt-in” procedure that their responses could be used for research purposes and linked to other data sources); (2) they completed Periodic Health Assessments in the year before their deployment and in the 15 months after their deployment; and (3) they completed the Post-Deployment Health Assessment in the month after their deployment. The University of Pennsylvania Institutional Review Board and a Department of Defense Human Research Protection Official reviewed and approved this study.

### Measures

#### Optimism

Four optimism items from the Global Assessment Tool, adapted from the revised Life Orientation Test,^34^ were asked on a 5-point Likert-type scale ranging from 1 (strongly disagree) to 5 (strongly agree). Items included the following: “In uncertain times, I usually expect the best”; “I rarely count on good things happening to me” (reverse scored); “Overall, I expect more good things to happen to me than bad”; and “If something can go wrong for me, it will” (reverse scored). Internal consistency was acceptable (α = .72). Continuous optimism represented the mean of the 4 items. In addition, soldiers were grouped into the following optimism tertiles: low (1.00-2.75), moderate (3.00-3.75), and high (4.00-5.00). Because of the distribution of optimism scores and an interest in ensuring that the low group actually reflected low levels of optimism, the low group contained a smaller percentage of the sample (8.0%) compared with the moderate (39.9%) and high (52.1%) groups.

#### New Pain

Pain reports were culled from soldiers’ postdeployment Periodic Health Assessment. The items asked “Do you or have you ever had [with separate responses for back pain, joint pain, and frequent headaches]” (yes or no)? We also created a composite measure that reflected any new back, joint, or headache pain (yes or no).

#### Combat Measures

Combat intensity assessed the occurrence (yes or no) of the following 5 potentially traumatic events during deployment: encountered dead bodies or saw people killed or wounded, felt in great danger of being killed, engaged in direct combat involving discharging a weapon, experienced a blast or explosion, and experienced a vehicular crash. Responses were summed, with higher scores indicating reporting more combat stressors. Combat injury (single item) assessed whether a soldier reported being wounded, injured, assaulted, or otherwise hurt during his or her deployment (yes or no). Combat intensity and combat injury, which were assessed on the Post-Deployment Health Assessment, were thought to serve as surrogates for PTSD and other psychiatric comorbidities and were expected to be strongly associated with greater odds of reporting new postdeployment pain.

#### Health Measures

Baseline chronic pain was culled from soldiers’ predeployment Periodic Health Assessment. Soldiers reported whether they currently had or had ever had chronic pain (yes or no). We adjusted for chronic pain at baseline because having an existing pain condition has long been associated with an increased likelihood of developing more painful conditions.^35,36,37^ Because smoking has been identified as a unique risk factor in chronic pain,^38,39^ nicotine status, which was obtained from soldiers’ predeployment Periodic Health Assessment, was included to capture whether soldiers reported smoking tobacco products, dipping, or chewing (yes or no).

#### Demographic and Military Characteristics

Demographic and military characteristics were obtained from Defense Manpower Data Center administrative records. Covariates included age (scaled in decades), sex (male vs female), race/ethnicity (non-Hispanic white vs other), marital status (married vs not married), educational attainment (up through high school vs more than high school), branch (active duty, Reserve, or National Guard), rank (officer vs enlisted), whether a soldier had previously deployed (yes vs no), and deployment location (Afghanistan or Iraq). Demographic characteristics (eg, race/ethnicity) were initially obtained through self-report, whereas military characteristics (eg, rank) were obtained through official records. Self-reported race/ethnicity information was dichotomized for the present analyses and was included to account for commonly observed differences.^40,41^

### Statistical Analysis

Analyses were performed in the Person-Event Data Environment between July 2016 and November 2018 using SAS Enterprise Guide (version 7.12; SAS Institute Inc). This study relied exclusively on existing, secondary Army data. We used binomial logistic regression to examine the association between continuous optimism and new pain (separate models examined any pain, back pain, joint pain, and frequent headaches), adjusting for covariates, which were entered into the model simultaneously. We repeated these analyses, replacing continuous optimism with optimism tertiles. To obtain all 3 pairwise comparisons, we first modeled high optimism as the reference group and then moderate optimism as the reference group. Multicollinearity diagnostics did not detect any problems.

Post hoc analyses tested for optimism by sex interactions and, separately, optimism by marital status interactions. Statistically significant interactions (2-sided *P* < .05) were followed up with stratified analyses to clarify the nature of the interaction.

To test for possible bias, we examined whether there were any systematic differences between soldiers who did and did not report back, joint, or headache pain at baseline (ie, excluded vs included). In addition, we compared soldiers in the analytic sample with soldiers who were excluded because of missing assessments.

## Results

### Participants

**Figure 1.**
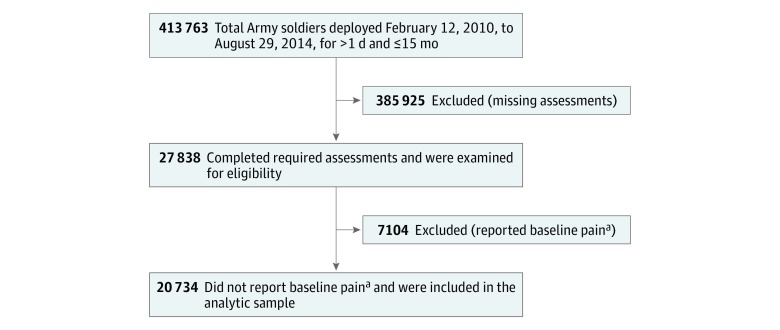
Flow Diagram of the Sample Selection ^a^Baseline pain refers to back pain, joint pain, or frequent headaches before deployment.

Of the 413 763 Army active duty, Reserve, and National Guard soldiers who deployed to Afghanistan or Iraq between February 12, 2010, and August 29, 2014 (>1 day and ≤15 months), 385 925 soldiers (93.3%) were missing 1 or more of the required assessment forms. Of the remaining 27 838 soldiers who were examined for eligibility, 7104 soldiers (25.5%) were excluded because they reported predeployment back pain, joint pain, or frequent headaches. These exclusions yielded a final analytic sample of 20 734 eligible soldiers. Figure 1 shows a flowchart of the sample selection in this study.

### Anticipating New Postdeployment Pain

Among 20 734 US Army soldiers (87.8% male; mean [SD] age, 29.06 [8.42] years), 37.3% reported pain in at least 1 new area of the body after deployment: 25.3% reported new back pain, 23.1% reported new joint pain, and 12.1% reported new frequent headaches. The results of McNemar tests indicated that new back pain and joint pain were reported more frequently than new frequent headaches. Approximately half of the sample (52.1%) reported high optimism, 39.9% reported moderate optimism, and 8.0% reported low optimism. Additionally, both stressful combat experiences (46.4%) and combat injuries (20.2%) were fairly common. A summary of sample characteristics is listed in Table 1.

**Table 1.** Distribution of Demographic, Military, and Health Covariates Among 20 734 US Army Soldiers

Variable	Value
Optimism, mean (SD)^a^	3.87 (0.75)
Age, mean (SD), y	29.06 (8.42)
Combat intensity, mean (SD)^b^	0.85 (1.13)
Sex, No. (%)	
Male	18 205 (87.80)
Female	2529 (12.20)
Race/ethnicity, No. (%)	
Non-Hispanic white	14 020 (67.62)
Nonwhite	6714 (32.38)
Marital status, No. (%)	
Married	10 464 (50.47)
Not married	10 270 (49.53)
Education, No. (%)	
≤High school	14 032 (67.68)
>High school	6702 (32.32)
Use tobacco, No. (%)	
No	13 759 (66.36)
Yes	6975 (33.64)
Predeployment chronic pain, No. (%)	
No	20 592 (99.32)
Yes	142 (0.68)
Branch, No. (%)	
Active duty	7491 (36.13)
Reserve	4390 (21.17)
National Guard	8853 (42.70)
Rank, No. (%)	
Officer	2993 (14.44)
Enlisted	17 741 (85.56)
Previously deployed, No. (%)	
No	12 711 (61.31)
Yes	8023 (38.69)
Deployment location, No. (%)	
Afghanistan	16 052 (77.42)
Iraq	4682 (22.58)
Injured while deployed, No. (%)	
No	16 555 (79.84)
Yes	4179 (20.16)

^a^ Optimism was adapted from the revised Life Orientation Test^34^ and assessed on a 5-point Likert-type scale ranging from 1 (strongly disagree) to 5 (strongly agree).

^b^ Combat intensity assessed the occurrence (yes or no) of the following 5 potentially traumatic events during deployment: encountered dead bodies or saw people killed or wounded, felt in great danger of being killed, engaged in direct combat involving discharging a weapon, experienced a blast or explosion, and experienced a vehicular crash. Responses were summed, with higher scores indicating reporting more combat stressors.

As a continuous measure, each 1-U increase in optimism was associated with 11% lower odds of reporting any new pain after deployment (odds ratio [OR], 0.89; 95% CI, 0.86-0.93) (Table 2). Examining the pain areas separately revealed that optimism was associated with 8% lower odds of developing new back pain (OR, 0.92; 95% CI, 0.88-0.96) and 8% lower odds of developing new joint pain (OR, 0.92; 95% CI, 0.88-0.96). However, we did not observe a significant statistical association between optimism and new frequent headaches after deployment (OR, 0.96; 95% CI, 0.91-1.02).

**Table 2.** Association Between Continuous Optimism and Incident Postdeployment Pain

Variable	Odds Ratio (95% CI)			
	Any New Pain	New Back Pain	New Joint Pain	New Frequent Headaches
Optimism	0.89 (0.86-0.93)	0.92 (0.88-0.96)	0.92 (0.88-0.96)	0.96 (0.91-1.02)
Age, in decades	1.35 (1.29-1.40)	1.18 (1.13-1.23)	1.41 (1.34-1.48)	1.13 (1.06-1.20)
Sex				
Male	1 [Reference]	1 [Reference]	1 [Reference]	1 [Reference]
Female	1.25 (1.14-1.36)	1.07 (0.97-1.19)	0.90 (0.81-1.01)	1.93 (1.71-2.17)
Race/ethnicity				
Non-Hispanic white	1 [Reference]	1 [Reference]	1 [Reference]	1 [Reference]
Nonwhite	1.06 (0.99-1.13)	1.13 (1.05-1.21)	1.07 (1.00-1.15)	1.26 (1.15-1.38)
Marital status				
Not married	1 [Reference]	1 [Reference]	1 [Reference]	1 [Reference]
Married	1.12 (1.05-1.20)	1.14 (1.06-1.22)	1.09 (1.01-1.17)	1.21 (1.10-1.33)
Education				
≤High school	1 [Reference]	1 [Reference]	1 [Reference]	1 [Reference]
>High school	0.98 (0.90-1.06)	1.03 (0.95-1.12)	0.96 (0.88-1.05)	0.88 (0.79-0.99)
Use tobacco				
No	1 [Reference]	1 [Reference]	1 [Reference]	1 [Reference]
Yes	1.16 (1.09-1.24)	1.16 (1.08-1.25)	1.14 (1.06-1.23)	1.02 (0.93-1.12)
Predeployment chronic pain				
No	1 [Reference]	1 [Reference]	1 [Reference]	1 [Reference]
Yes	2.51 (1.76-3.58)	1.65 (1.17-2.34)	3.04 (2.16-4.27)	1.48 (0.94-2.33)
Branch				
Active duty	1 [Reference]	1 [Reference]	1 [Reference]	1 [Reference]
Reserve	0.76 (0.70-0.82)	0.88 (0.80-0.97)	0.77 (0.69-0.84)	0.98 (0.86-1.11)
National Guard	0.93 (0.87-1.00)	1.12 (1.04-1.21)	1.10 (1.02-1.19)	1.44 (1.30-1.59)
Rank				
Enlisted	1 [Reference]	1 [Reference]	1 [Reference]	1 [Reference]
Officer	0.80 (0.73-0.89)	0.80 (0.72-0.90)	0.85 (0.76-0.96)	0.68 (0.58-0.79)
Previously deployed				
No	1 [Reference]	1 [Reference]	1 [Reference]	1 [Reference]
Yes	1.08 (1.01-1.15)	1.08 (1.00-1.16)	1.04 (0.97-1.12)	0.99 (0.90-1.09)
Deployment location				
Afghanistan	1 [Reference]	1 [Reference]	1 [Reference]	1 [Reference]
Iraq	0.82 (0.76-0.88)	0.77 (0.72-0.84)	0.74 (0.68-0.81)	0.67 (0.60-0.75)
Combat intensity	1.14 (1.11-1.17)	1.11 (1.08-1.14)	1.08 (1.05-1.12)	1.14 (1.10-1.18)
Injured while deployed				
No	1 [Reference]	1 [Reference]	1 [Reference]	1 [Reference]
Yes	2.15 (2.01-2.32)	1.79 (1.66-1.93)	1.95 (1.81-2.11)	1.64 (1.49-1.81)

We next modeled optimism tertiles (Table 3) and found that compared with soldiers with high optimism (lowest odds of new pain) soldiers with low optimism had the following characteristics: 35% greater odds of any new pain (OR, 1.35; 95% CI, 1.21-1.50), 30% greater odds of new back pain (OR, 1.30; 95% CI, 1.16-1.46), 21% greater odds of new joint pain (OR, 1.21; 95% CI, 1.07-1.38), and 18% greater odds of new frequent headaches (OR, 1.18; 95% CI, 1.01-1.38). In addition, we observed a larger increase in odds of new pain when comparing the moderate-optimism and low-optimism groups rather than the high-optimism and moderate-optimism groups.

**Table 3.** Association Between Optimism (Tertiles) and Incident Postdeployment Pain^a^

Variable	Odds Ratio (95% CI)			
	Any New Pain	New Back Pain	New Joint Pain	New Frequent Headaches
Optimism				
Moderate (reference is high)	1.11 (1.04-1.18)	1.04 (0.98-1.12)	1.07 (1.00-1.15)	1.02 (0.93-1.11)
Low (reference is high)	1.35 (1.21-1.50)	1.30 (1.16-1.46)	1.21 (1.07-1.38)	1.18 (1.01-1.38)
Low (reference is moderate)	1.21 (1.09-1.36)	1.24 (1.10-1.40)	1.14 (1.00-1.29)	1.16 (0.99-1.36)
Age, in decades	1.34 (1.29-1.40)	1.18 (1.12-1.23)	1.41 (1.34-1.47)	1.13 (1.06-1.20)
Sex				
Male	1 [Reference]	1 [Reference]	1 [Reference]	1 [Reference]
Female	1.24 (1.14-1.36)	1.07 (0.97-1.18)	0.90 (0.81-1.01)	1.93 (1.71-2.17)
Race/ethnicity				
Non-Hispanic white	1 [Reference]	1 [Reference]	1 [Reference]	1 [Reference]
Nonwhite	1.06 (0.99-1.13)	1.13 (1.05-1.21)	1.07 (1.00-1.15)	1.26 (1.15-1.38)
Marital status				
Not married	1 [Reference]	1 [Reference]	1 [Reference]	1 [Reference]
Married	1.12 (1.05-1.20)	1.14 (1.06-1.22)	1.09 (1.01-1.17)	1.21 (1.10-1.33)
Education				
≤High school	1 [Reference]	1 [Reference]	1 [Reference]	1 [Reference]
>High school	0.98 (0.91-1.06)	1.03 (0.95-1.12)	0.96 (0.88-1.05)	0.89 (0.79-0.99)
Use tobacco				
No	1 [Reference]	1 [Reference]	1 [Reference]	1 [Reference]
Yes	1.16 (1.09-1.24)	1.16 (1.08-1.25)	1.14 (1.06-1.23)	1.02 (0.93-1.12)
Predeployment chronic pain				
No	1 [Reference]	1 [Reference]	1 [Reference]	1 [Reference]
Yes	2.51 (1.76-3.57)	1.65 (1.16-2.34)	3.04 (2.16-4.27)	1.48 (0.94-2.33)
Branch				
Active duty	1 [Reference]	1 [Reference]	1 [Reference]	1 [Reference]
Reserve	0.76 (0.70-0.82)	0.88 (0.80-0.97)	0.76 (0.69-0.84)	0.98 (0.86-1.11)
National Guard	0.93 (0.87-1.00)	1.12 (1.04-1.21)	1.10 (1.02-1.19)	1.44 (1.30-1.59)
Rank				
Enlisted	1 [Reference]	1 [Reference]	1 [Reference]	1 [Reference]
Officer	0.80 (0.72-0.89)	0.80 (0.71-0.89)	0.85 (0.76-0.96)	0.68 (0.58-0.79)
Previously deployed				
No	1 [Reference]	1 [Reference]	1 [Reference]	1 [Reference]
Yes	1.08 (1.01-1.16)	1.08 (1.00-1.16)	1.04 (0.97-1.13)	0.99 (0.90-1.09)
Deployment location				
Afghanistan	1 [Reference]	1 [Reference]	1 [Reference]	1 [Reference]
Iraq	0.82 (0.76-0.88)	0.77 (0.71-0.84)	0.74 (0.68-0.81)	0.70 (0.60-0.75)
Combat intensity	1.14 (1.11-1.17)	1.11 (1.08-1.14)	1.08 (1.05-1.12)	1.14 (1.09-1.18)
Injured while deployed				
No	1 [Reference]	1 [Reference]	1 [Reference]	1 [Reference]
Yes	2.15 (2.00-2.31)	1.78 (1.65-1.92)	1.95 (1.80-2.11)	1.64 (1.49-1.81)

^a^ This model was run with high optimism as the reference group and again with moderate optimism as the reference group to obtain all 3 pairwise comparisons. However, this did not change any of the estimates for the covariates.

Associations between demographic characteristics and new pain are summarized in Table 2 and Table 3. Being older and being married were associated with increased odds of reporting new postdeployment pain. Women were more likely to report any new pain or new frequent headaches; for women and men, reporting chronic pain at baseline was associated with greater odds of reporting any new pain, new back pain, or new joint pain after deployment. In addition, being injured while deployed and reporting stressful combat experiences were each associated with greater odds of reporting new pain after deployment. In contrast, being an officer (vs an enlisted soldier) and deploying to Iraq (vs Afghanistan) were each associated with reduced odds of reporting new pain after deployment. Of the soldiers who reported new pain in all 3 areas, a disproportionate number (63.3%) were National Guard soldiers compared with active duty (19.7%) and Reserve (17.0%) soldiers.

### Tests of Moderation

**Figure 2.**
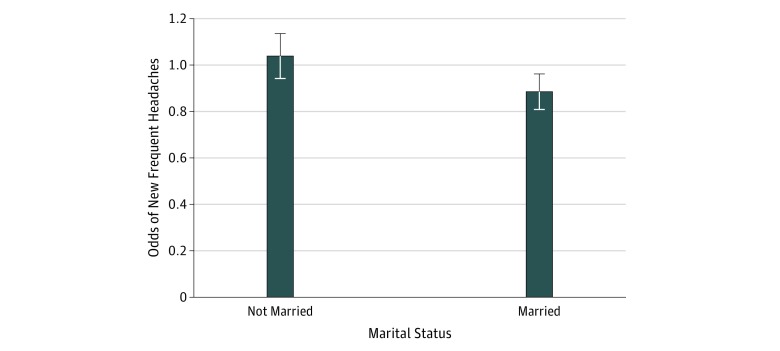
Optimism and Odds of Reporting New Frequent Headaches by Marital Status The odds ratio for not married was 1.05 (95% CI, 0.96-1.14); married, 0.89 (95% CI, 0.83-0.97). Error bars indicate 95% CIs.

We also tested interactions between optimism and sex and, separately, between optimism and marital status. We did not observe any statistically significant interactions between optimism and sex or between optimism and marital status in relation to any new pain, new back pain, or new joint pain. However, we observed a statistically significant interaction between optimism and marital status in association with new frequent headaches (OR, 0.85; 95% CI, 0.76-0.95). Stratified analyses (Figure 2) revealed that greater optimism was associated with lower odds of reporting frequent headaches after deployment among married soldiers (OR, 0.89; 95% CI, 0.83-0.97) but not unmarried soldiers (OR, 1.05; 95% CI, 0.96-1.14).

### Sample Generalization

Compared with soldiers in the analytic sample (N = 20 734), soldiers excluded for reporting predeployment back pain, joint pain, or frequent headaches (n = 7104) were slightly less optimistic (3.87 vs 3.72, respectively) and older (29.1 vs 33.8 years, respectively). Soldiers who reported predeployment pain were more likely to be married (70.4% vs 50.5%), have more than a high school education (42.1% vs 32.3%), report predeployment chronic pain (16.5% vs 0.7%), be active duty (58.5% vs 36.1%), or have previously deployed (70.7% vs 38.7%) compared with soldiers in the analytic sample. In addition, soldiers with predeployment pain were less likely than those in the analytic sample to be in the National Guard (24.7% vs 42.7%) or be enlisted (79.9% vs 85.7%). All other demographic differences were negligible (<5% difference). In addition, a comparison between the analytic sample and the sample excluded because of missing assessments revealed that, aside from differences in branch (active duty soldiers were more likely to be missing assessments), the 2 groups were fairly comparable.

## Discussion

Few studies have assessed instances of new pain in military personnel after deployment. In the present study of 20 734 Army soldiers who deployed to Afghanistan or Iraq, 37.3% reported experiencing at least 1 new area of pain after deployment, with new back pain (25.3%) and new joint pain (23.1%) described more often than new frequent headaches (12.1%). A major strength of this study was the large sample size, which enabled us to obtain accurate point estimates and narrow 95% CIs. In addition, although only a subset of the population completed the health assessments required for inclusion in this study, the study sample was fairly representative of the 2010 Army active duty population^42^ (sample vs population): officers (14.4% vs 16.8%), female (12.2% vs 13.4%), 25 years or younger (45.0% vs 41.2%), and married (50.5% vs 58.7%).

In regard to optimism, the soldiers were remarkably optimistic, with 52.1% reporting high levels of optimism before deployment. Furthermore, greater predeployment optimism was associated with significantly lower odds of reporting new postdeployment pain (particularly any, back, and joint pain), even while adjusting for other important pain-related factors, such as combat intensity, combat injuries, baseline chronic pain, nicotine use, and key demographic and military characteristics. As such, every 1-U increase in optimism was independently associated with 11% lower odds of reporting new pain in any of the 3 bodily areas evaluated. Moreover, the least optimistic soldiers had 35% greater odds of reporting a new instance of pain compared with those with the highest levels of optimism. The difference between the soldiers with moderate levels of optimism and those with the highest levels was not, for the most part, statistically significant, suggesting that extremely high levels of optimism may not be necessary to experience benefit.

Similar to prior research,^6^ we found that being older and being married were associated with reporting pain after deployment. Furthermore, we observed a number of associations between military characteristics and odds of reporting new postdeployment pain. For example, enlisted soldiers were more likely to report new postdeployment pain compared with officers. This may in part be because in the combat theater, senior officers (and some noncommissioned officers) are typically responsible for strategic planning, whereas enlisted soldiers are typically responsible for riskier and more physically demanding tasks (eg, combat patrols, convoy operations, and ensuring that routes are clear of improvised explosive devices). In addition, compared with active duty soldiers, National Guard soldiers had a higher odds of reporting new postdeployment back pain, joint pain, or frequent headaches, whereas Reserve soldiers had lower odds of reporting new pain in these areas. This may in part be because National Guard soldiers tend to engage in direct combat, whereas Reserve soldiers tend to perform combat support and service duties (less risky assignments), and active duty soldiers serve in a wide variety of roles, including direct combat, combat support, and combat service.

This study also revealed that soldiers who deployed to Iraq had a reduced odds of reporting new pain relative to soldiers who deployed to Afghanistan. During the study deployment time frame (February 12, 2010, to August 29, 2014), walking patrols were fairly common. Poor infrastructure in Afghanistan (eg, lack of good roads and walking paths), coupled with extreme temperatures, may explain in part the more frequent new pain reported by soldiers who deployed to Afghanistan compared with Iraq.

In regard to combat experiences, we found that combat injuries and stressful combat events were common (20.2% and 46.4%, respectively) and associated with new postdeployment pain. That combat experiences were determinants of new pain has been previously shown^43,44,45^ and is intuitive (physical injury and acute pain can transition into chronic pain), while PTSD and other psychiatric comorbidities are common correlates of pain.^46^ Herein, combat experiences were used as a surrogate for PTSD and other psychiatric comorbidities because these traumatic experiences typically precede the manifestation of psychiatric sequelae.^16^ Despite the robust association between the onset of new postdeployment pain and key demographic, military, and combat experiences, predeployment optimism remained significantly associated with new postdeployment pain.

No studies to date have explored the prospective association between optimism and postdeployment pain in military personnel. Although data from the Global Assessment Tool have been shown to differentiate Army Rangers from non-Rangers (Rangers demonstrate greater optimism, engagement, and organizational trust and lower levels of depression, catastrophizing, and loneliness),^47^ the postdeployment associations with optimism have yet to be reported. Others have shown that optimism is associated with better health outcomes in diverse patient populations, including cardiovascular disease,^23^ diabetes,^48^ and even mortality from multiple causes.^49^ Therefore, the protective association between optimism and pain is not surprising given that high optimism has been associated with decreased pain and better quality of life in civilian populations when pain is present.^27,28^ Herein, we add the protective association between optimism and the development of pain in soldiers after deployment.

Optimism is generally considered a trait, although it has been estimated that optimism is only about 25% heritable.^50^ Therefore, optimism can be learned and is thus a modifiable factor.^51^ Previous studies^52,53,54^ have shown that straightforward interventions can result in higher levels of optimism. For example, interventions can include imagining and writing about a vision of one’s best possible self (ie, the person we would like to be)^52,53^ or imagery training to increase positive appraisals of ambiguous social situations as opposed to anticipating the worst possible outcome.^54^ Furthermore, interventions that promote the expression of gratitude and teach meditation and mindfulness practices, as well as more structured interventions like cognitive behavior therapy that more directly challenge catastrophic thinking, can promote optimism.^55^

### Limitations

This study has some limitations. First, the assessment of new areas of pain was limited by the number of areas that were assessed both before and after deployment. The 3 areas selected are those where pain is commonly observed but by no means were exhaustive; therefore, our rates of new pain may underestimate the true number of soldiers with new postdeployment pain. Second, neither the duration nor the intensity of pain was assessed; therefore, the overall influence, chronicity, and severity of the pain are not known. Third, this study only examined reports of new pain within 15 months of returning from deployment. The extent to which optimism is associated with pain over a longer follow-up remains unknown. Fourth, as with all research, we were only able to examine soldiers who completed the necessary assessments and allowed their responses to be used for research purposes. However, the study sample was representative of the 2010 Army active duty population.^42^ Fifth, we did not adjust for psychiatric disorders, such as PTSD, in the present analyses. However, we adjusted for potentially traumatic experiences during deployment, which have consistently been linked to PTSD,^56,57,58^ along with comorbid psychiatric disorders, such as depression and anxiety disorders.^59,60,61^

## Conclusions

Reducing instances of new pain after deployment is critical because 37.3% of soldiers herein reported at least 1 new area of pain. Over and above other common determinants of pain after deployment, including demographic and military characteristics and combat experiences, higher levels of optimism were associated with lower odds of reporting new pain. Data from Army psychological assessments like the Global Assessment Tool could be used to identify soldiers with low levels of optimism who may benefit from programs geared toward enhancing optimism. In current and future conflicts, these strategies could help diminish the consequences of pain, one of the most common and costly outcomes of deployment.
